# Efficacy and safety of polyethylene glycol loxenatide in treating mild-to-moderate diabetic kidney disease in type 2 diabetes patients: a randomized, open-label, clinical trial

**DOI:** 10.3389/fendo.2024.1387993

**Published:** 2024-07-19

**Authors:** YongSheng Cao, Shujie Cao, Jiangang Zhao, Jianqin Zhao, Yanan Zhao, Ying Liu

**Affiliations:** ^1^ Department of Neurology, Sunshine Union Hospital, Weifang, Shandong, China; ^2^ Department of Endocrinology, Sunshine Union Hospital, Weifang, Shandong, China

**Keywords:** dapagliflozin, diabetic kidney disease, glomerular filtration rate, lipid profiles, polyethylene glycol loxenatide, type 2 diabetes mellitus, urinary albumin-to-creatinine ratio

## Abstract

**Objective:**

This study aimed to evaluate the efficacy and safety of polyethylene glycol loxenatide (PEG-Loxe) compared to those of dapagliflozin in patients with mild-to-moderate diabetic kidney disease (DKD), a prevalent microvascular complication of type 2 diabetes mellitus (T2DM). The study is set against the backdrop of increasing global diabetes incidence and the need for effective DKD management.

**Methods:**

This study constituted a single-center, randomized, open-label, clinical trial. The trial included patients with mild-to-moderate DKD and suboptimal glycemic control. Eligible participants were randomly allocated to one of the two groups for treatment with either PEG-Loxe or dapagliflozin. The primary endpoint was the change in UACR from baseline at 24 weeks.

**Results:**

Overall, 106 patients were randomized and 80 patients completed the study. Following 24 weeks of treatment, the PEG-Loxe group exhibited a mean percent change in baseline UACR of −29.3% (95% confidence interval [CI]: −34.8, −23.7), compared to that of −31.8% in the dapagliflozin group (95% CI: −34.8, −23.7). Both PEG-Loxe and dapagliflozin showed similar efficacy in reducing UACR, with no significant difference between the groups (*p* = 0.336). The HbA1c levels decreased by −1.30% (95% CI: −1.43, −1.18) in the PEG-Loxe group and by −1.29% (95% CI: −1.42, −1.17) in the dapagliflozin group (*p* = 0.905). The TG levels decreased by −0.56 mmol/L (95% CI: −0.71, −0.42) in the PEG-Loxe group and −0.33 mmol/L (95% CI: −0.48, −0.19) in the dapagliflozin group (*p* = 0.023). Differences in TC, HDL-C, LDL-C, SBP, and DBP levels between the groups were not statistically significant (all *p* > 0.05). Safety profiles were consistent with previous findings, with gastrointestinal adverse events being more common in the PEG-Loxe group.

**Conclusions:**

PEG-Loxe is as effective as dapagliflozin in improving urine protein levels in patients with mild-to-moderate DKD and offers superior benefits in improving lipid profiles. These findings support the use of PEG-Loxe in DKD management, contributing to evidence-based treatment options.

**Clinical Trial Registration:**

www.chictr.org.cn, identifier ChiCTR2300070919.

## Introduction

In recent years, the incidence of diabetes has steadily risen, affecting 540 million adults globally, with more than 90% diagnosed with type 2 diabetes mellitus (T2DM) (1). Diabetes mellitus results in a range of macrovascular and microvascular complications, posing significant health risks (2). Diabetic kidney disease (DKD) represents one such microvascular complication of diabetes mellitus (3). Approximately 20%–40% of individuals with diabetes develop DKD (4).

DKD constitutes a chronic kidney disease characterized by a complex pathogenesis (5). Clinically, it is marked by persistent albuminuria and/or a gradual decline in the glomerular filtration rate (GFR), eventually leading to end-stage renal disease (ESRD) (6). Besides being a primary cause of ESRD, DKD significantly elevates the risk of cardiovascular events and all-cause mortality in individuals with T2DM (7).

Recently, advancements in drug therapy for DKD have emerged from research into the pathogenic mechanisms of diabetes mellitus, its complications, and the introduction of new drug classes. One notable drug is GLP-1RA, which induces hypoglycemic effects by boosting insulin secretion and suppressing glucagon secretion (8, 9), while also ameliorating lipid and blood pressure levels (10, 11). Research indicates that this drug class markedly lowers the risk of kidney-related composite endpoints, such as progression to macroalbuminuria, doubling of serum creatinine, ESRD, and kidney disease-related mortality, compared to those by placebo (12, 13).

Polyethylene glycol loxenatide (PEG-Loxe), approved in 2019, is a once-weekly GLP-1RA formulation derived from amino acid and polyethylene glycol (PEG) modifications of exendin-4 (14). A prior phase 3 study reported that PEG-Loxe therapy, either alone or combined with metformin for 24 weeks in T2DM patients, showed promising efficacy and safety, evidenced by reductions of 1.14%–1.34% in hemoglobin A1c (HbA1c) levels and 10.3%–25.0% in gastrointestinal adverse event (AE) rates (15, 16). Additionally, a randomized-controlled trial demonstrated that 16 weeks of PEG-Loxe treatment led to an average weight loss of 7.52 kg in T2DM patients that were also overweight or obese (17). However, the efficacy and safety of PEG-Loxe in DKD patients remain unreported and unknown. Thus, this study aimed to assess the efficacy and safety of PEG-Loxe in patients with mild-to-moderate DKD.

## Methods

### Study design

This study constituted a single-center, randomized, open-label, clinical trial. Approval for the study was granted by the Institutional Review Board of Sunshine Union Hospital (IRB no.: YGRHLLKY-0005). Prior to inclusion in the study, all patients provided informed consent. Conducted in alignment with the Declaration of Helsinki and Good Clinical Practice, the study was registered with the Chinese Clinical Trial Registry (ChiCTR) (no.: ChiCTR2300070919).

### Study participants

The inclusion criteria included the following:

- Written informed consent.
- Age ≥ 18 years with a clinical diagnosis of T2DM.
- HbA1c between 7.0% and 10.0%.
- estimated glomerular filtration rate (eGFR) ranging from 30 to 90 mL/min/1.73 m^2^.
- Urinary albumin-to-creatinine ratio (UACR) >30 mg/g and ≤5,000 mg/g.
- Agreement by women to use contraception during the intervention.

The exclusion criteria included the following:

- History of diabetic ketoacidosis or type 1 diabetes mellitus.
- Severe cardiovascular, cerebrovascular, or hepatic disease.
- Renal transplant recipients or patients on dialysis therapy.
- Allergy or intolerance to GLP-1RA.
- Use of GLP-1RA or SGLT2 inhibitors within 12 weeks before study enrollment.
- Severe gastrointestinal disorders.
- History of pancreatitis.

### Randomization and masking

Eligible participants were randomly allocated to one of the two groups for treatment with either PEG-Loxe or dapagliflozin. The randomization was conducted by a statistician using a computer-generated random number sequence, assigning patients to the groups in a 1:1 ratio. During data analysis, statisticians were blinded to the group assignments of the participants.

### Procedures

Each patient’s existing treatment regimen was supplemented with either PEG-Loxe or dapagliflozin. PEG-Loxe was administered subcutaneously once weekly. The treatment adhered to a fixed-dose escalation schedule, starting with an initial dose of 0.1 mg for 4 weeks, followed by a maintenance dose of 0.2 mg until study completion. Likewise, dapagliflozin was initiated at 5 mg for 2 weeks, followed by a maintenance dose of 10 mg until the study’s conclusion.

Patients were subjected to follow-up examinations at 12 and 24 weeks, encompassing physical examinations and data collection. Demographic data, vital signs, and laboratory results were systematically recorded. Systolic and diastolic blood pressure (SBP and DBP, respectively) measurements were taken using a sphygmomanometer (HBP-1300, OMRON, Dalian, China). Morning fasting blood samples were collected and analyzed in the Laboratory Department of Sunshine Union Hospital. Laboratory examination encompassed UACR, 24-h urine protein, eGFR, HbA1c, fasting plasma glucose (FPG), and a blood lipid panel. Furthermore, all AEs were recorded.

### Outcomes

The primary endpoint focused on the change in UACR from baseline till after 24 weeks of treatment. Secondary endpoints encompassed 24-h urine protein, eGFR, HbA1c, FPG, body weight, total cholesterol (TC), triglycerides (TG), low-density lipoprotein cholesterol (LDL-C), high-density lipoprotein cholesterol (HDL-C), SBP, and DBP after 24 weeks of treatment.

### Statistical analysis

The primary outcome assessed was the non-inferiority of PEG-Loxe compared that of to dapagliflozin in terms of UACR change from baseline. A sample size of 64 offered an 80% power to affirm the non-inferiority of UACR change (35% margin), presuming a geometric mean coefficient of variation of 66% (18) and a bilateral α level of 0.05. The withdrawal rate was established at 20%, resulting in a total sample size of 80.

To evaluate the primary endpoint, a mixed model for repeated measures (MMRM) was employed to compare changes in log-transformed UACR (log UACR) between the two groups. In this model, treatment group, time, and their interaction were considered fixed effects, with baseline log UACR as a covariate. MMRM analysis was also applied to 24-h urine protein, eGFR, HbA1c, FPG, TC, TG, LDL-C, HDL-C, SBP, and DBP. Patients lacking UACR data at 6 months were excluded from the analysis due to the inability to calculate change values. Differences yielding a *p-*value < 0.05 were deemed statistically significant. Statistical analyses were conducted using SAS v. 9.4 software.

## Results

**Figure 1:**
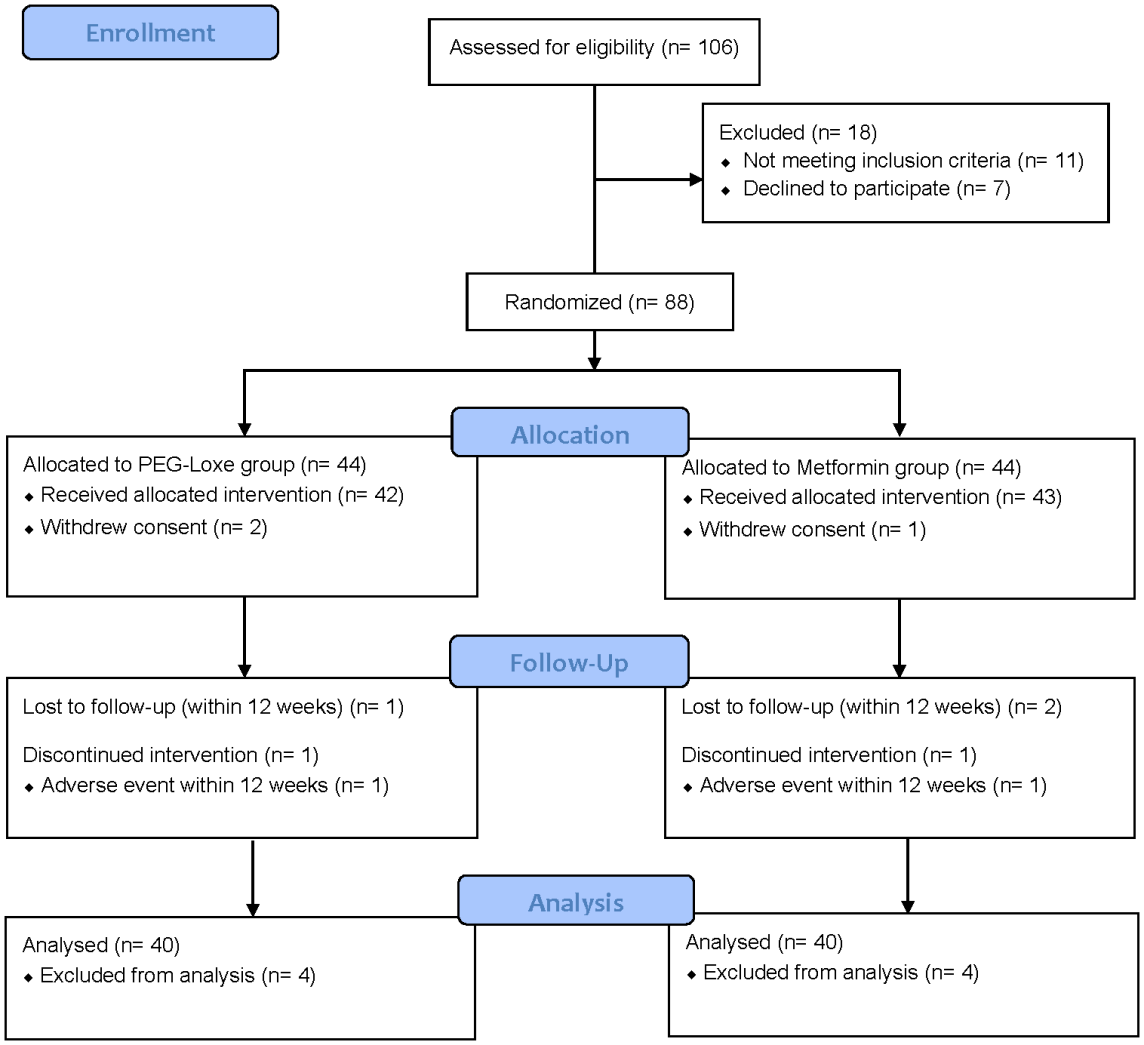
Consort flow diagram.

Between April 2023 and July 2023, 106 participants were evaluated for eligibility, with 88 ultimately being included in the study. Throughout the study, three participants withdrew consent, two exited due to AEs, and three were lost to follow-up, culminating in 80 participants completing the study (**Figure 1**). **Table 1** displays the baseline characteristics of both groups. Except for those in LDL-C, there were no statistically significant differences in any parameters between the groups.

**Table 1:** Baseline characteristics.

	PEG-Loxe (n=40)	Dapagliflozin (n=40)	*P* value
Women, N (%)	22 (55.0)	17 (42.5)	0.263
Age, y	51.0 (9.3)	48.4 (10.1)	0.230
Duration, y	7.8 (3.9)	7.3 (3.7)	0.515
Body weight, kg	75.6 (10.6)	77.2 (8.1)	0.436
BMI, kg/m^2^	26.4 (1.4)	26.3 (1.6)	0.765
HbA1c, %	8.31 (0.71)	8.31 (0.77)	0.976
FPG, mmol/L	9.45 (1.02)	9.58 (1.19)	0.616
UACR, mg/g	108.5 (79.3-150.8)	106.5 (77.0-197.3)	0.965
eGFR, ml/min/1.73 m^2^	68.2 (9.3)	67.9 (12.3)	0.902
24-hour urinary protein, mg	222.5 (64.5)	238.1 (96.3)	0.398
TC, mmol/L	5.90 (1.15)	5.73 (1.07)	0.498
TG, mmol/L	2.85 (0.65)	2.88 (0.76)	0.876
LDL-C, mmol/L	3.69 (0.48)	3.50 (0.35)	0.048
HDL-C, mmol/L	0.93 (0.15)	0.93 (0.12)	0.954
SBP, mmHg	137.1 (13.6)	135.7 (14.1)	0.653
DBP, mmHg	96.7 (10.5)	94.3 (11.8)	0.340
Antidiabetic drugs, N (%)			
Biguanides	20 (50.0)	23 (57.5)	0.501
SU/glinides	12 (30.0)	13 (32.5)	0.809
AGI	23 (57.5)	20 (50.0)	0.501
TZD	12 (30.0)	18 (45.0)	0.166
Insulin	20 (50.0)	20 (50.0)	1.000
ACEI/ARB, N (%)	19 (47.5)	18 (45.0)	0.823
Diuretics, N (%)	3 (7.5)	2 (5.0)	0.644
Lipid-lowering agent, N (%)	19 (47.5)	23 (57.5)	0.371

BMI, body mass index; HbA1c, glycated hemoglobin; FPG, fasting plasma glucose; UACR, urinary albumin-to-creatinine ratio; eGFR, estimated glomerular filtration rate; TC, total cholesterol; TG, triglycerides; LDL-C, low-density lipoprotein cholesterol; HDL-C, high-density lipoprotein cholesterol; SBP, systolic blood pressure; DBP, diastolic blood pressure; SU, Sulfonylurea; AGI, alpha-glucosidase inhibitor; TZD, thiazolidinedione; ACEI/ARB, angiotensin-converting enzyme inhibitors/Angiotension II receptor blockers. Data are mean (SD) or median (interquartile range).

Initially, the median UACR stood at 108.5 mg/g in the PEG-Loxe group (interquartile range [IQR]: 79.3–150.8) and 106.5 mg/g in the dapagliflozin group (IQR: 77.0–197.3). Following 24 weeks of treatment, the PEG-Loxe group exhibited a mean percent change in baseline UACR of −29.3% (95% confidence interval [CI]: −34.8, −23.7), compared to that of −31.8% in the dapagliflozin group (95% CI: −34.8, −23.7). The intergroup difference was 2.6% (95% CI: −5.3, −10.4), a non-statistically significant variation (*p* = 0.336). After 24 weeks of treatment, the change in eGFR was 6.3 mL/min/1.73 m^2^ (4.4, 8.2) in the PEG-Loxe group and 7.2 mL/min/1.73 m^2^ (5.3, 9.1) in the dapagliflozin group. The difference between the groups was −0.9 mL/min/1.73 m^2^ (−3.6, 1.8), which was not statistically significant (*p* = 0.504). The intergroup difference in 24-h urine protein was −0.6 mg (−19.2, 18.1; *p* = 0.953) (**Table 2**).

**Table 2:** Primary and secondary endpoints at week 24.

	PEG-Loxe (n=40)	Dapagliflozin (n=40)	Between-Group Difference (95% CI)	*P* value
UACR, mg/g				
Baseline	108.5 (79.3-150.8)	106.5 (77.0-197.3)		
Week 24	75.0 (57.3-119.3)	76.5 (41.0-153.8)		
Change from baseline (%) (95% CI)	-29.3 (-34.8, -23.7)	-31.8 (-34.8, -23.7)	2.6 (-5.3, -10.4)	0.336
eGFR, ml/min/1.73 m^2^				
Baseline	68.2 (9.3)	67.9 (12.3)		
Week 24	74.4 (10.9)	75.0 (12.6)		
Change from baseline (%) (95% CI)	6.3 (4.4, 8.2)	7.2 (5.3, 9.1)	-0.9 (-3.6, 1.8)	0.504
24-hour urinary protein, mg				
Baseline	222.5 (64.5)	238.1 (96.3)		
Week 24	182.2 (63.1)	195.1 (88.8)		
Change from baseline (%) (95% CI)	-41.9 (-55.1, -28.8)	-41.4 (-54.6, -28.2)	-0.6 (-19.2, 18.1)	0.953
HbA1c, %				
Baseline	8.31 (0.71)	8.31 (0.77)		
Week 24	7.01 (0.50)	7.01 (0.69)		
Change from baseline (%) (95% CI)	-1.30 (-1.43, -1.18)	-1.29 (-1.42, -1.17)	-0.01 (-0.19, 0.17)	0.905
FPG, mmol/L				
Baseline	9.45 (1.02)	9.58 (1.19)		
Week 24	7.23 (0.58)	7.50 (0.82)		
Change from baseline (%) (95% CI)	-2.26 (-2.45, -2.08)	-2.04 (-2.22, -1.85)	-0.23 (-0.49, 0.03)	0.083
Body weight, kg				
Baseline	75.6 (10.6)	77.2 (8.1)		
Week 24	71.8 (9.3)	74.1 (7.9)		
Change from baseline (%) (95% CI)	-3.9 (-4.7, -3.1)	-3.1 (-3.9, -2.3)	-0.8 (-1.9, 0.3)	0.151
TC, mmol/L				
Baseline	5.90 (1.15)	5.73 (1.07)		
Week 24	4.79 (1.01)	4.85 (0.93)		
Change from baseline (%) (95% CI)	-1.08 (-1.30, -0.87)	-0.92 (-1.14, -0.70)	-0.16 (-0.47, 0.15)	0.299
TG, mmol/L				
Baseline	2.85 (0.65)	2.88 (0.76)		
Week 24	2.29 (0.73)	2.54 (0.87)		
Change from baseline (%) (95% CI)	-0.56 (-0.71, -0.42)	-0.33 (-0.48, -0.19)	-0.23 (-0.44, -0.02)	0.032
LDL-C, mmol/L				
Baseline	3.69 (0.48)	3.50 (0.35)		
Week 24	2.80 (0.58)	2.69 (0.60)		
Change from baseline (%) (95% CI)	-0.86 (-1.03, -0.68)	-0.86 (-1.03, -0.68)	0.00 (-0.25, 0.25)	0.989
HDL-C, mmol/L				
Baseline	0.93 (0.15)	0.93 (0.12)		
Week 24	0.97 (0.12)	1.00 (0.11)		
Change from baseline (%) (95% CI)	0.05 (0.02, 0.07)	0.07 (0.05, 0.09)	-0.02 (-0.06, 0.01)	0.179
SBP, mmHg				
Baseline	137.1 (13.6)	135.7 (14.1)		
Week 24	129.6 (9.17)	131.4 (10.7)		
Change from baseline (%) (95% CI)	-7.2 (-9.1, -5.4)	-4.6 (-6.5, -2.7)	-2.6 (-5.3, 0.0)	0.053
DBP, mmHg				
Baseline	96.7 (10.5)	94.3 (11.8)		
Week 24	91.3 (8.1)	90.4 (9.4)		
Change from baseline (%) (95% CI)	-4.4 (-6.2, -2.5)	-4.3 (-6.1, -2.5)	-0.1 (-2.7, 2.5)	0.956

BMI, body mass index; HbA1c, glycated hemoglobin; FPG, fasting plasma glucose; UACR, urinary albumin-to-creatinine ratio; eGFR, estimated glomerular filtration rate; TC, total cholesterol; TG, triglycerides; LDL-C, low-density lipoprotein cholesterol; HDL-C, high-density lipoprotein cholesterol; SBP, systolic blood pressure; DBP, diastolic blood pressure. UACR has been log-transformed for statistical analysis and back-transformed for presentation.

Following 24 weeks of treatment, HbA1c levels decreased by −1.30% (−1.43, −1.18) in the PEG-Loxe group and by −1.29% (−1.42, −1.17) in the dapagliflozin group (*p* = 0.905), while FPG levels decreased by −2.26 mmol/L (−2.45, −2.08) in the PEG-Loxe group and −2.04 mmol/L (−2.22, −1.85) in the dapagliflozin group (*p* = 0.083). The weight change difference between the PEG-Loxe and dapagliflozin groups was −0.8 kg (−1.9, 0.3), lacking statistical significance (*p* = 0.151) (**Table 2**).

Regarding blood lipids and blood pressure, TG levels decreased by −0.56 mmol/L (−0.71, −0.42) in the PEG-Loxe group and −0.33 mmol/L (−0.48, −0.19) in the dapagliflozin group. The intergroup difference was −0.23 mmol/L (−0.44, −0.02) (*p* = 0.023). Differences in TC, HDL-C, LDL-C, SBP, and DBP levels between the groups were not statistically significant (all *p* > 0.05) (**Table 2**).**Table 3** presents efficacy indicators at 12 weeks of treatment, mirroring those observed at 24 weeks. In terms of safety, the drug was generally well-tolerated, with AEs aligning with expectations. Gastrointestinal AEs occurred more frequently with PEG-Loxe, while urinary tract infections were more prevalent with dapagliflozin (**Table 4**).

**Table 3:** Efficacy endpoints at week 12.

	PEG-Loxe (n=40)	Dapagliflozin (n=40)	Between-Group Difference (95% CI)	*P* value
UACR, mg/g				
Baseline	108.5 (79.3-150.8)	106.5 (77.0-197.3)		
Week 12	86.0 (68.8-122.5)	87.5 (54.3-166.3)		
Change from baseline (%) (95% CI)	-18.3 (-21.7, -23.7)	-20.1 (-16.7, -23.4)	1.7 (-3.0, 6.5)	0.380
eGFR, ml/min/1.73 m^2^				
Baseline	68.2 (9.3)	67.9 (12.3)		
Week 12	72.0 (10.7)	72.9 (12.4)		
Change from baseline (%) (95% CI)	3.9 (2.5, 5.2)	5.0 (3.7, 6.4)	-1.2 (-3.1, 0.8)	0.227
24-hour urinary protein, mg				
Baseline	222.5 (64.5)	238.1 (96.3)		
Week 12	194.4 (58.0)	207.3 (78.0)		
Change from baseline (%) (95% CI)	-29.8 (-38.1, -21.6)	-29.1 (-37.3, -20.8)	-0.8 (-12.5, 10.9)	0.895
HbA1c, %				
Baseline	8.31 (0.71)	8.31 (0.77)		
Week 12	7.34 (0.58)	7.42 (0.80)		
Change from baseline (%) (95% CI)	-0.94 (-1.06, -0.82)	-0.89 (-1.01, -0.77)	-0.06 (-0.23, 0.11)	0.512
FPG, mmol/L				
Baseline	9.45 (1.02)	9.58 (1.19)		
Week 12	7.92 (0.64)	7.98 (0.85)		
Change from baseline (%) (95% CI)	-1.57 (-1.76, -1.38)	-1.57 (-1.76, -1.38)	-0.01 (-0.28, 0.26)	0.962
Body weight, kg				
Baseline	75.6 (10.6)	77.2 (8.1)		
Week 12	73.3 (10.0)	75.3 (7.8)		
Change from baseline (%) (95% CI)	-2.4 (-2.9, -1.8)	-1.9 (-2.5, -1.4)	-0.5 (-1.2, 0.3)	0.244
TC, mmol/L				
Baseline	5.90 (1.15)	5.73 (1.07)		
Week 12	5.09 (1.11)	5.08 (0.92)		
Change from baseline (%) (95% CI)	-0.79 (-0.96, -0.63)	-0.679 (-0.83, -0.51)	-0.12 (-0.36, 0.11)	0.291
TG, mmol/L				
Baseline	2.85 (0.65)	2.88 (0.76)		
Week 12	2.42 (0.69)	2.59 (0.78)		
Change from baseline (%) (95% CI)	-0.43 (-0.55, -0.32)	-0.29 (-0.40, -0.17)	-0.15 (-0.31, 0.02)	0.080
LDL-C, mmol/L				
Baseline	3.69 (0.48)	3.50 (0.35)		
Week 12	3.10 (0.50)	2.99 (0.46)		
Change from baseline (%) (95% CI)	-0.57 (-0.69, -0.45)	-0.54 (-0.66, -0.42)	-0.02 (-0.20, 0.15)	0.784
HDL-C, mmol/L				
Baseline	0.93 (0.15)	0.93 (0.12)		
Week 12	0.95 (0.14)	0.98 (0.14)		
Change from baseline (%) (95% CI)	0.03 (0.00, 0.05)	0.05 (0.03, 0.08)	-0.03 (-0.06, 0.01)	0.157
SBP, mmHg				
Baseline	137.1 (13.6)	135.7 (14.1)		
Week 12	132.1 (11.1)	132.5 (12.3)		
Change from baseline (%) (95% CI)	-5.0 (-6.5, -3.4)	-3.3 (-4.9, -1.7)	-1.6 (-3.9, 0.6)	0.149
DBP, mmHg				
Baseline	96.7 (10.5)	94.3 (11.8)		
Week 12	93.7 (8.7)	92.5 (10.0)		
Change from baseline (%) (95% CI)	-2.7 (-4.1, -1.3)	-2.1 (-3.5, -0.7)	-0.6 (-2.6, 1.3)	0.521

BMI, body mass index; HbA1c, glycated hemoglobin; FPG, fasting plasma glucose; UACR, urinary albumin-to-creatinine ratio; eGFR, estimated glomerular filtration rate; TC, total cholesterol; TG, triglycerides; LDL-C, low-density lipoprotein cholesterol; HDL-C, high-density lipoprotein cholesterol; SBP, systolic blood pressure; DBP, diastolic blood pressure. UACR has been log-transformed for statistical analysis and back-transformed for presentation.

**Table 4:** Summary of safety.

	PEG-Loxe, No. (%) (n=40)	Dapagliflozin, No. (%) (n=40)
Any AE	8 (20.0)	7 (17.5)
Any SAE		
Death	0 (0)	0 (0)
Other	1 (2.5)	1 (2.5)
AE by severity		
Severe	1 (2.5)	2 (5.0)
Moderate	2 (5.0)	1 (2.5)
Mild	5 (12.5)	4 (19.2)
Discontinuation because of AEs	1 (2.5)	1 (2.5)
AEs reported in ≥5% of patients by SOC/PT		
Gastrointestinal disorders	7 (17.5)	1 (2.5)
Nausea	4 (10.0)	0 (0)
Vomiting	1 (2.5)	0 (0)
Diarrhea	2 (5.0)	1 (2.5)
Infections and infestations	0 (0)	5 (9.6)
Urinary tract infection	0 (0)	5 (9.6)
Hypoglycemia	1 (2.5)	1 (2.5)
Level 1	1 (2.5)	1 (2.5)
Level 2	0 (0)	0 (0)
Level 3	0 (0)	0 (0)

AE, adverse event; SAE, serious adverse event; SOC, system organ class; PT, preferred term.

## Discussion

To the best of our knowledge, this study presents the first direct comparison of PEG-Loxe and dapagliflozin in patients with mild-to-moderate DKD. This single-center, randomized, open-label clinical trial demonstrated that PEG-Loxe’s efficacy in reducing proteinuria was comparable to that of dapagliflozin in patients with mild-to-moderate DKD. Furthermore, PEG-Loxe also offered the advantage of enhancing blood lipid levels.

GLP-1 RA, a novel class of glucose-lowering agents, has demonstrated cardiorenal protection in T2DM patients in trials focusing on cardiovascular outcomes (19–21). Clinical guidelines endorse GLP-1 RA as the primary glucose-lowering agents for treating T2DM patients with concurrent atherosclerotic cardiovascular disease and as a secondary option for DKD treatment (1, 22). A previous study reported that 24 weeks of liraglutide treatment led to a 13.2% reduction in UACR in T2DM patients with a baseline UACR of 24.8 mg/g (23). For T2DM patients with concurrent moderate-to-severe chronic kidney disease, 26 weeks of dulaglutide treatment resulted in a 27.7% decrease in UACR (24). In T2DM patients with a baseline UACR of 135 mg/g, a 26-week semaglutide treatment regimen led to a 14% reduction in UACR (25). In this study, the baseline UACR in the PEG-Loxe group was 108.5 mg/g, and it decreased by 29.3% after 6 months of treatment, indicating renoprotective effects of PEG-Loxe akin to other GLP-1 RAs.

SGLT2 inhibitors (SGLT2i), such as dapagliflozin, are universally recommended as first-line therapeutic agents in DKD treatment, supported by large-scale studies demonstrating cardiac and renal benefits (26–29). A meta-analysis encompassing 16 clinical studies with T2DM patients having a baseline eGFR of 30–90 mL/min/1.73 m^2^ revealed that over 2 years, SGLT2i reduced UACR by 17%–33%, while GLP-1 RA achieved a 19%–22% reduction. Overall, the effect of SGLT2i in reducing urine protein levels was comparable to or marginally more potent than that of GLP-1 RA (30). This study also involved patients with a baseline eGFR of 30–90 mL/min/1.73 m^2^, where 6 months of treatment with dapagliflozin and PEG-Loxe led to UACR reductions of 31.8% and 29.3%, respectively, aligning with previous findings that PEG-Loxe and dapagliflozin have comparable efficacy in improving urine protein levels. This study suggests that PEG-Loxe may be considered for patients with mild-to-moderate DKD if SGLT2i is insufficient to meet glycemic targets or not tolerated.

Regarding glycemic control, a meta-analysis of 315 trials (involving 35,022 patients) revealed no significant disparity in the efficacy of GLP-1 RA and SGLT2i in reducing HbA1c levels; however, GLP-1 RA was more effective in lowering fasting glucose levels than SGLT2i (31). GLP-1 RA regulates blood glucose levels via various mechanisms, such as by enhancing insulin secretion and inhibiting glucagon secretion in a glucose concentration-dependent manner, alongside delaying gastric emptying and diminishing food intake through central appetite suppression (32, 33). This multifaceted approach might account for GLP-1 RA’s superior glycemic control compared to that by SGLT2i’s singular glucose-lowering mechanism SGLT2i. While the reduction in FPG levels in the PEG-Loxe group was not significantly different from that in the SGLT2i group in this study, PEG-Loxe seemed to marginally outperform dapagliflozin in lowering FPG.

Both GLP-1 RA and SGLT2i are effective in improving lipid levels (34, 35). A meta-analysis comprising 25 studies with 1,595 non-alcoholic fatty liver disease patients showed that GLP-1RA reduced TG levels more effectively than SGLT2i; however, the effect on TC, HDL-C, and LDL-C levels was similar for both (36). The current study yielded similar findings in patients with mild-to-moderate DKD. Compared to that by dapagliflozin, PEG-Loxe exhibited a more pronounced reduction in TG, with no significant differences in TC, HDL-C, and LDL-C levels between the groups. This finding indicates that PEG-Loxe surpasses dapagliflozin in improving lipid levels in patients with mild-to-moderate DKD.

Prior to this study, the safety of PEG-Loxe in treating patients with mild-to-moderate DKD was not reported. In this study, the AEs in the PEG-Loxe group were mainly gastrointestinal, with no unexpected AEs being noted. This observation aligns with previous safety outcomes observed in T2DM patients and those with T2DM that are also overweight or obese (16, 17), suggesting that PEG-Loxe can be safely used to treat patients with mild-to-moderate DKD.

The study has the following limitations. First, this study assessed only the short-term efficacy results over 24 weeks, without assessing long-term efficacy. Second, the sample size was small, which necessitates validation of the findings with larger cohorts in future studies. Third, the single-center, open-label design of the study could have potentially introduced reporting bias.

In conclusion, the findings of this randomized clinical trial demonstrated that the efficacy of PEG-Loxe in reducing urine protein in mild-to-moderate DKD patients was akin to that of dapagliflozin, with the added benefit of improving lipid levels. These findings offer significant evidence-based research for the use of PEG-Loxe in DKD patients.

## Data availability statement

The raw data supporting the conclusions of this article will be made available by the authors, without undue reservation.

## Ethics statement

The studies involving humans were approved by the Institutional Review Board of Sunshine Union Hospital. The studies were conducted in accordance with the local legislation and institutional requirements. The participants provided their written informed consent to participate in this study.

## Author contributions

YC: Writing – original draft, Writing – review & editing. SC: Data curation, Writing – original draft, Writing – review & editing. JGZ: Data curation, Writing – original draft, Writing – review & editing. JQZ: Data curation, Writing – original draft, Writing – review & editing. YZ: Data curation, Writing – original draft, Writing – review & editing. YL: Methodology, Project administration, Writing – original draft, Writing – review & editing.
